# Influence of Preoperative Perforator Mapping Modality on Outcomes in DIEP Flap Breast Reconstruction: A Multicenter Registry Analysis

**DOI:** 10.3390/jcm15197562

**Published:** 2026-09-29

**Authors:** Uwe von Fritschen, Andreas Arkudas, Maike Keck, Sonia Fertsch, Emre Gazyakan, Daniel Schiltz

**Affiliations:** 1 Department of Plastic and Aesthetic Surgery, Hand Surgery, Helios Hospital Emil von Behring, Walterhöferstraße 11, 14165 Berlin, Germany; daniel.schiltz@helios-gesundheit.de; 2 Department of Plastic, Hand and Reconstructive Surgery, University Hospital Regensburg, 93053 Regensburg, Germany; 3 Department of Plastic and Hand Surgery, University Hospital Erlangen, Friedrich-Alexander-University Erlangen-Nürnberg (FAU), 91054 Erlangen, Germany; andreas.arkudas@uk-erlangen.de; 4 Department of Plastic, Reconstructive and Aesthetic Surgery, Agaplesion Diakonieklinikum Hamburg, 20259 Hamburg, Germany; keck@aesthetik-kaifu.de; 5 Department of Plastic and Aesthetic Surgery, Interdisciplinary Breast Center, Sana Kliniken Düsseldorf GmbH, 40625 Düsseldorf, Germany; sonia.fertsch@sana.de; 6 Faculty of Health, University of Witten-Herdecke, 58455 Witten, Germany; 7 Department of Hand, Plastic and Reconstructive Surgery, Burn Center, BG Klinik Ludwigshafen, Heidelberg University, 67071 Ludwigshafen, Germany; emre.gazyakan@bgu-ludwigshafen.de

**Keywords:** DIEP, deep inferior epigastric perforator flap, breast reconstruction, imaging

## Abstract

**Background**: Preoperative perforator mapping is widely used to guide perforator selection in deep inferior epigastric perforator (DIEP) flap breast reconstruction. However, the impact of different imaging modalities on operative variables and postoperative outcomes remains unclear. **Methods**: We performed a retrospective analysis of a multicenter breast reconstruction registry including patients who underwent autologous DIEP flap breast reconstruction between 2011 and 2018. Mapping modalities included computed tomography angiography (CTA), magnetic resonance angiography (MRA), color-coded duplex sonography (CCDS), hand-held Doppler sonography (HHDS) or no preoperative mapping. Outcomes included operative time, number of included perforators, and major flap-related complications. Subgroup analyses were performed for unilateral procedures and Holm zone classifications. Associations between perforator number, body mass index (BMI), and complication risk were evaluated. **Results**: A total of 4283 DIEP flaps were included for analysis. CTA was associated with fewer included perforators and a lower rate of major complications in unilateral reconstructions compared with other modalities. CCDS was associated with the highest number of included perforators. An increasing perforator number was not associated with reduced major complication rates. Higher BMI was associated with increased complication risk, particularly in flaps including Holm zone 4. **Conclusions**: Preoperative CTA-based perforator mapping was associated with fewer selected perforators and lower major complication rates in unilateral DIEP reconstruction. The number of included perforators alone did not predict complications, while obesity appeared to increase risk in extended perfusion territories. These findings support individualized preoperative planning and careful consideration of patient risk factors.

## 1. Introduction

Breast cancer remains the most common malignancy among women worldwide [1]. Improvements in diagnostics and oncologic treatment have led to increasing survival rates and a growing demand for breast reconstruction following mastectomy [2,3].

While implant-based breast reconstruction is still the most commonly used technique [4,5], autologous breast reconstruction using the deep inferior epigastric perforator (DIEP) flap has become a widely accepted standard technique [6,7,8,9,10,11,12,13,14].

The complication rate has decreased steadily with an average flap loss rate of less than 3% [15]. Successful DIEP flap reconstruction depends on adequate flap perfusion, which is determined by the quality and location of perforating vessels from the deep inferior epigastric system. Traditionally, perforators were identified intraoperatively, which could increase operative time and introduce uncertainty in perforator selection.

Several imaging techniques are now used for preoperative perforator mapping, including computed tomography angiography (CTA), magnetic resonance angiography (MRA), color-coded duplex sonography (CCDS) and hand-held Doppler sonography (HHDS). These modalities allow surgeons to visualize the vascular anatomy prior to surgery and may improve surgical planning. Despite their widespread adoption, the extent to which mapping modality influences operative decisions and postoperative outcomes remains incompletely understood.

The present study analyzes data from a large multicenter registry [16] to evaluate whether preoperative perforator mapping modality is associated with operative characteristics and clinical outcomes in DIEP flap breast reconstruction. While portions of the registry have been reported previously [17,18,19,20,21,22], this aspect has not been addressed. The objectives of this study were to evaluate the association between preoperative perforator mapping modality and operative variables such as operative time and number of included perforators, to assess whether perforator number is associated with major complications, and to analyze the influence of body mass index (BMI) and Holm zone classification on complication risk.

## 2. Materials and Methods

This retrospective study used data from a multicenter prospective registry of autologous breast reconstruction procedures. The structure, objectives, and certification criteria of the registry have been described in detail elsewhere [16].

Patients undergoing DIEP flap breast reconstruction between 2011 and 2018 were included. Procedures were categorized according to the preoperative perforator mapping modality used: CTA, MRA, CCDS, HHDS or no mapping.

Recorded variables included, among others, patient demographics, BMI, operative time, number of included perforators, and postoperative complications. Major flap-related complications were defined as events leading to partial or complete flap loss. Wound healing disorder, hematoma, revision of the anastomosis and infection were defined as minor complication. At the flap level (n = 4283), total flap loss occurred in 88 flaps (2.1%) and partial flap loss meeting the major-complication threshold in 46 flaps (1.1%) overall (total + partial = 134, matching the overall major complication count in Table 1; a further 29 flaps had a documented partial loss that did not meet this threshold and were not classified as major complications. By mapping modality and subgroup, the corresponding total/partial flap loss counts are given in the two rightmost columns of Table 1.

BMI categories were defined according to the World Health Organization (WHO) classification as underweight (<18.5), normal weight (18.5–24.9), overweight (25.0–29.9) and obesity grade I–III (≥30.0). If patients underwent more than one mapping modality, analysis was based on a hierarchy of methods assigning patients to the higher ranked modality—MRA, CTA, CCDS, HHDS and no mapping (NONE), chosen because it approximates decreasing anatomical resolution of the compared modalities, so that patients undergoing more than one study are conservatively assigned to the most informative modality actually available to the surgeon. If “not obtained” was recorded in all method categories, the variable was regarded missing. Of 3653 patients with valid preoperative-diagnostics data, 535 (14.6%) underwent more than one modality (533 with two, 2 with three); a sensitivity analysis restricted to the core registry years 2012–2017 (n = 3284 DIEP flaps) showed a modality distribution consistent with the full 2011–2018 cohort.

### 2.1. Patient Cohort

In total, 4844 patients underwent unilateral or bilateral microsurgical breast reconstruction in 22 centers, comprising 4940 surgeries (n = 4209 unilateral; n = 731 simultaneous bilateral) and corresponding to 5671 microsurgical flaps performed and registered. Data were pooled across these 22 participating centers with heterogeneous case volumes and institutional imaging protocols; center-level clustering was not formally modeled, and center-specific volumes are not reported due to data-sharing agreements with participating institutions, which represents a potential source of residual confounding.

Exclusion criteria were other than DIEP flap in unilateral surgeries (n = 1045), or in bilateral surgeries (n = 47; 94 flaps), inclusion of perforators from both sides of the DIEP flap and inclusion of both pedicles in the flap (n = 44), use of the superficial inferior epigastric vein (SIEV, n = 119) and BMI smaller than 18.5 kg/m^2^ (n = 52) because these small subgroups showed insufficient statistical power.

**Figure 1:**
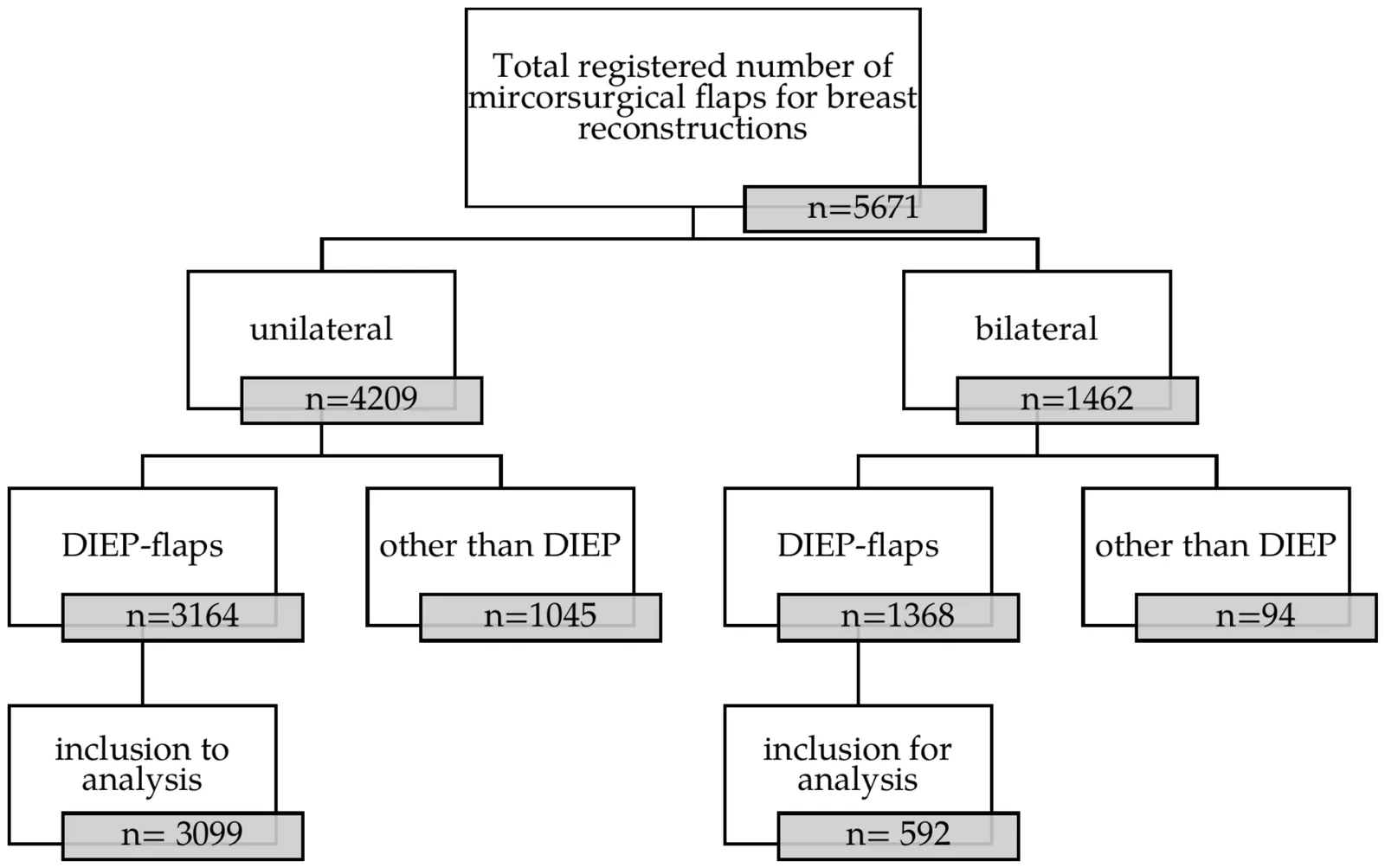
Flowchart of final number of flaps included for analysis. (Other flaps were: free transverse rectus abdominis muscle flap (TRAM), inferior gluteal artery perforator flap (I-GAP), superior gluteal artery perforator flap (S-GAP), fasciocutaneous inferior gluteal artery flap (FCI) and thoracodorsal artery perforator flap (TDAP)).

Finally, 3099 unilateral and 592 simultaneous bilateral microsurgical breast reconstructions (total flap number of n = 4283) using a DIEP flap were included in the final data set for statistical analysis (Figure 1 and Table 2). For bilateral reconstructions, each flap was analyzed as an independent observation in flap-level analyses. Patient-level variables (e.g., BMI, comorbidities) were shared across both flaps of a bilateral pair, which represents a potential source of non-independence. This limitation is acknowledged, and sensitivity analyses in the unilateral subgroup are reported throughout. Because bilateral flaps from the same patient are likely to be more similar to each other than to flaps from different patients, formal significance levels in flap-level bilateral analyses may be modestly overstated; the consistency of findings with the independent unilateral subgroup analyses (Section 3.3) supports the robustness of the main conclusions.

### 2.2. Statistical Analysis

Statistical analyses were performed by the Center for Clinical Studies, University Hospital Regensburg, Germany, using SPSS version 29.0.1. All tests were two-sided, and statistical significance was set at *p* < 0.05. All analyses were considered exploratory; therefore, no adjustment for multiple testing was applied. Associations between imaging modality, number of included perforators, body mass index (BMI), and major complication risk were evaluated. Subgroup analyses were performed for unilateral reconstructions and according to perfusion territory based on the Holm zone classification. Bonferroni correction (n_comp = 10) was applied to the pairwise post-hoc comparisons of perforator number across modalities (Section 3.3); all other reported comparisons, including the modality-specific odds ratios for major complications and the BMI-by-Holm-zone subgroup analysis, were not corrected for multiple testing and should be regarded as exploratory. Under a conservative Bonferroni correction applied across the total number of comparisons reported in this manuscript, the association between CT angiography, reduced major complications (Total cohort: odds ratio (OR) = 0.494, *p* = 0.0065; Unilateral: OR = 0.511, *p* = 0.0236) and all perforator-number pairwise comparisons except HHDS vs. None remain statistically significant; the BMI-by-Holm-zone-4 interaction was not re-verified against a corrected threshold in the present analysis and should be interpreted as hypothesis-generating.

### 2.3. Ethics

All elements of the study were performed in accordance with institutional guidelines and regulations. Ethical board approval was obtained from the Bavarian State Medical Association (156/17 S) and the Berlin Chamber of Physicians (Eth-V-Q/17). Study design is a pseudonymized retrospective multicenter cohort study.

## 3. Results

A total of 3691 patients that had 4283 DIEP flap reconstructions (3099 unilateral flaps and 2 × 592 simultaneous bilateral flaps) were included in the analysis (Table 3). CTA was the most frequently used imaging modality, followed by HHDS, CCDS and without preoperative imaging. A subset of procedures was performed by MRA (Table 4).

### 3.1. Distribution of Preoperative Mapping Modalities (2011–2018)

**Figure 2:**
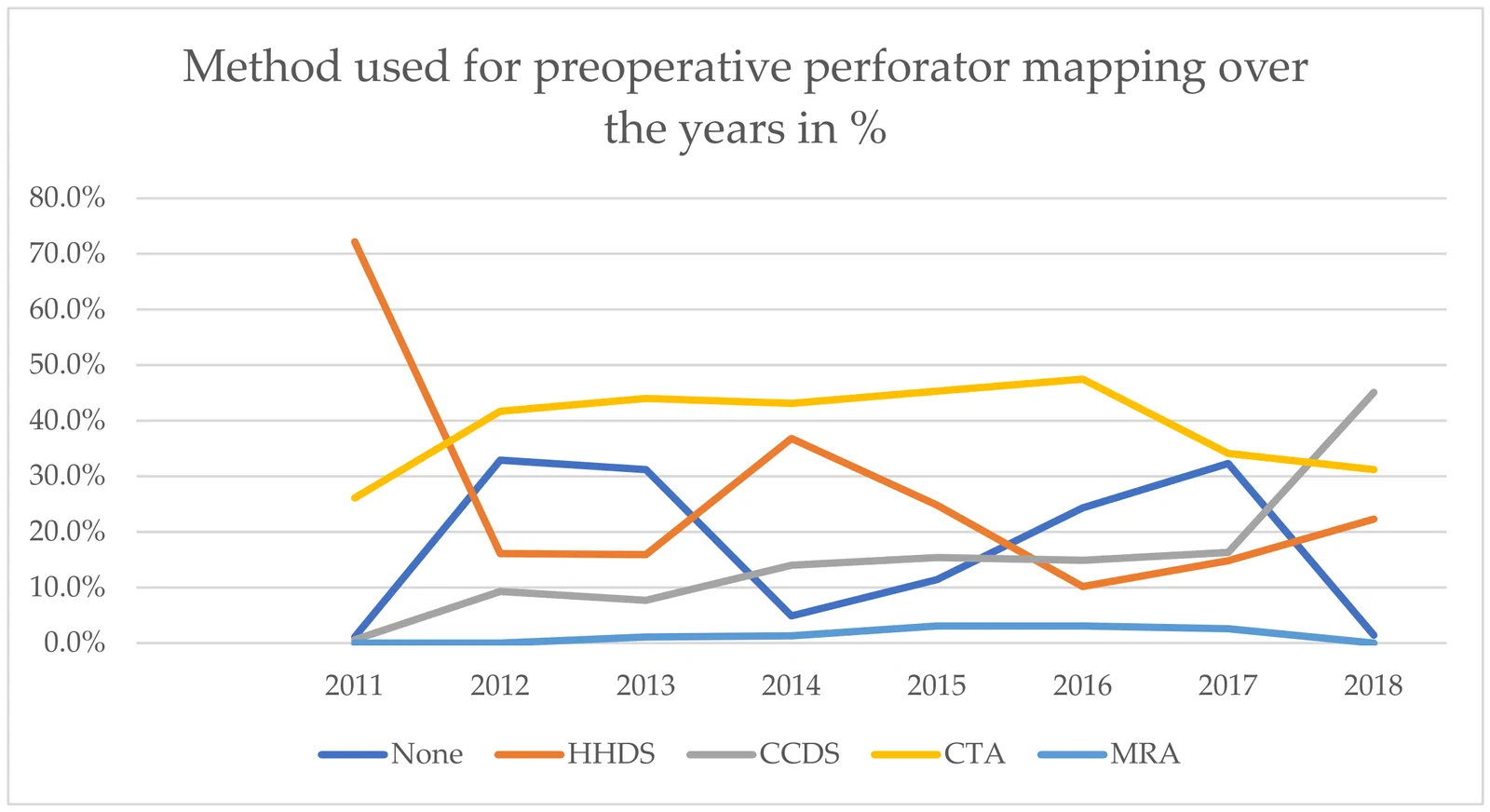
Percentage of use of each mapping method over the years.

Modalities were assigned by hierarchy (MRA > CTA > CCDS > HHDS > None). Missing values were excluded. Flap type and preoperative mapping modality were recorded as independent fields; the “None” mapping category reflects the absence of any preoperative vascular mapping. Over the observation period, CTA use declined while CCDS increased, reflecting a shift toward radiation-free imaging (Table 4 and Figure 2). The number of valid modality records per year ranged from n = 176 (2011) to n = 678 (2016) and n = 215 (2018), broadly tracking overall registry volume, which grew from 183 DIEP flaps in 2011 (the registry’s founding year) to a peak of 682 in 2016 before declining to 224 in 2018, the final year of data collection; a sensitivity analysis restricted to the core years 2012–2017 (n = 3284 DIEP flaps) showed a similar modality distribution and trend, indicating that this pattern is not an artifact of partial-year enrollment at the registry’s start and end.

### 3.2. Effect of Preoperative Mapping Modality on Operative Duration and Major Complications

Across the entire cohort (n = 3653 surgeries with valid data), the most frequently used preoperative mapping modality was CT angiography (CTA; 41.4%), followed by hand-held Doppler sonography (HHDS; 22.3%), no mapping (20.1%), color-coded duplex sonography (CCDS; 14.5%), and MR angiography (MRA; 1.8%). The distribution was comparable in the unilateral subgroup (CTA 41.3%, HHDS 22.6%, none 20.5%, CCDS 13.8%, MRA 1.7%) and the bilateral subgroup (CTA 41.6%, HHDS 20.6%, none 18.1%, CCDS 17.6%, MRA 2.0%). Over the observation period, a notable temporal trend was observed: the use of CTA declined while CCDS increased, reflecting a gradual shift toward radiation-free mapping techniques.

All mapping groups had significantly longer operative times than the No-mapping reference (analysis of variance (ANOVA) and Kruskal–Wallis both *p* < 0.001, across all cohorts) (Table 1).

Pairwise post-hoc comparisons demonstrated that CTA-based planning was associated with the selection of fewer perforators compared with other modalities. Despite the lower perforator count, CTA cases demonstrated lower rates of major complication rates in unilateral reconstructions (total 2.0%; unilateral 1.8%) and was the only modality with a statistically significant odds ratio vs. None (Total: OR = 0.494, *p* = 0.007; Unilateral: OR = 0.511, *p* = 0.024). In the bilateral subgroup, no significant difference in major complications was found across modalities (χ^2^ *p* = 0.130). Across all modalities, surgical duration was significantly longer than in the None group (ANOVA and Kruskal–Wallis both *p* < 0.001; e.g., CT angiography vs. None: 338.4 vs. 232.4 min, total cohort), a difference that likely reflects patient and procedural selection rather than a direct effect of imaging modality itself (see Discussion).

CCDS-guided planning was associated with the highest number of included perforators. However, an increasing perforator number was not associated with a reduction in major complication rates.

### 3.3. Influence of Preoperative Mapping Modality on Number of Included Perforators

Preoperative mapping modality had a highly significant effect on the number of perforators included in the DIEP flap (Kruskal–Wallis test: total H = 460.4, *p* < 0.001; unilateral H = 413.0, *p* < 0.001; bilateral combined H = 92.9, *p* < 0.001).

Pairwise post-hoc comparisons (Mann–Whitney U, Bonferroni correction, n_comp = 10) revealed consistent patterns across all subgroups. CCDS was associated with the highest number of included perforators (mean 2.55 ± 0.87), followed by HHDS (2.15 ± 1.00), no mapping (2.09 ± 0.71), CTA (1.73 ± 0.87), and MRA (1.47 ± 0.87). Pairwise post-hoc comparisons revealed that CCDS differed significantly from all other modalities (all *p* < 0.001 after Bonferroni correction). CTA and MRA were associated with significantly fewer included perforators than HHDS and no mapping (both *p* < 0.001). The only non-significant pairwise comparison was between no mapping and HHDS (*p* = 1.000 in all subgroups). The MR angiography subgroup was comparatively small (n = 64 total, n = 52 unilateral); comparisons involving this group should therefore be interpreted with caution (see Limitations).

CTA vs. MRA reached significance in the total and unilateral cohorts (*p* = 0.027 and *p* = 0.040, respectively) but not in the bilateral subgroup. Results were consistent across unilateral, bilateral side 1, bilateral side 2, and combined bilateral analyses.

### 3.4. Number of Perforators, Holm Zones, and Major Complications

To investigate whether a higher number of included perforators reduces major complication risk at equivalent flap complexity, unilateral cases were stratified by Holm perfusion zones: Group 1 (Holm zones 1–2, n = 445), Group 2 (Holm zone 1–3, n = 2354), and Group 3 (Holm zone 1–4, n = 250). A total of 50 cases were excluded due to missing data (n analysed = 3049).

Major complication rates were 2.9% in Group 1, 2.9% in Group 2, and 4.0% in Group 3. Within none of the groups did the number of included perforators significantly differ between patients with and without major complications (Group 1: mean 2.15 vs. 2.31 perforators, U = 3.230, *p* = 0.331; Group 2: mean 1.95 vs. 1.90, U = 74.020, *p* = 0.355; Group 3: mean 2.43 vs. 2.70, U = 1.292, *p* = 0.663).

In Groups 1 and 3, the mean perforator count was slightly higher in patients with major complications, although this trend did not reach statistical significance. Overall, a higher number of included perforators does not reduce major complication risk within any Holm zone group (Table 5).

### 3.5. Influence of BMI on Major Complication Rate Within Holm Zone Groups

Patients were further stratified by BMI group (<25, 25–30, >30 kg/m^2^) within each Holm zone group to examine the interaction between body composition and perfusion territory complexity (n analysed = 3034; 65 cases excluded due to missing data).

In Holm groups 1–3, BMI had no significant influence on major complication rate (Chi^2^ *p* = 0.339 and *p* = 0.472, respectively), with complication rates remaining stable across BMI subgroups.

In contrast, within Holm zone 4, BMI had a significant effect on complication risk (Chi^2^ = 11.791, *p* = 0.003). Major complication rates increased from 0.8% (BMI < 25), 4.3% (BMI 25–30), and 13.5% (BMI > 30). Post-hoc Fisher’s exact testing showed a significantly increased odds ratio for BMI > 30 compared with BMI < 25 (OR = 18.4, *p* = 0.003). No significant differences were observed between the BMI < 25 and BMI 25–30 (*p* = 0.171) or BMI 25–30 and BMI > 30 (*p* = 0.117).

Perforator count did not significantly predict major complications within BMI subgroups. Across all analyses, the number of included perforators was not associated with major complication risk, whereas BMI emerged as a relevant risk factor specifically in patients with the most extensive perfusion territory (Holm zone 4).

## 4. Discussion

This multicenter registry analysis demonstrates that preoperative perforator mapping modality influences perforator selection in DIEP flap breast reconstruction and may affect clinical outcomes. CTA-based perforator mapping may facilitate more selective perforator choice and was associated with fewer included perforators and lower major flap-related complication rates in unilateral DIEP flap reconstructions. In contrast, CCDS-guided planning resulted in higher perforator counts without reduction in complication rates. Additionally, obesity emerged as an important risk modifier, particularly in flaps including Holm zone 4.

A central finding of the present study is that the choice of preoperative imaging modality significantly influences the number of perforators ultimately included in the flap. CTA was associated with the lowest perforator counts and the small MRA subgroup showed the same trend, whereas CCDS and HHDS resulted in higher numbers. This likely reflects the detailed three-dimensional anatomical information provided by CTA, allowing surgeons to confidently select dominant perforators [23,24,25]. In contrast, although CCDS accurately localizes perforators, its more limited visualization of the intramuscular perforator course may encourage inclusion of additional perforators as a safety margin [26]. These findings are consistent with Rozen et al., who demonstrated that CTA provides superior visualization of deep inferior epigastric artery (DIEA) branching patterns and perforator course, enabling targeted dissection and selective perforator use [25].

Despite the lower perforator count, CTA-based planning was associated with the lowest major complication rate in unilateral reconstructions. Previous studies have similarly suggested that perforator quality and anatomic dominance may be more relevant than absolute perforator number alone [23,24,25,26,27,28,29,30,31,32]. However, this interpretation could not be directly tested, as detailed perforator characteristics (e.g., caliber, dominance) were not captured in this registry.

These findings are consistent with previous reports demonstrating improved outcomes with CTA-guided DIEP flap planning [23,24,27,33]. Alonso-Burgos et al. reported fewer complications requiring revision surgery in CTA-guided cases, while Fitzgerald O’Connor et al. observed a trend toward reduced complications with CTA [24,27]. The absence of a significant difference in bilateral reconstructions in the present study may reflect smaller flap size or limited statistical power in this subgroup.

Evidence regarding perforator number remains context-dependent. In DIEP-only analyses stratified by Holm zones, perforator count was not associated with reduced major complication rates. Contralateral perforators and SIEV drainage procedures were not included in these subgroup analyses because of limited case numbers. Because the present analyses are observational and subject to indication bias, perforator number should be interpreted primarily as a surrogate of flap design, procedural complexity, and intraoperative decision-making rather than an independent causal factor.

The longer operative duration observed in mapped cases likely reflects differences in patient selection, increased procedural complexity, and institutional practices rather than an imaging modality itself. Consequently, operative duration should not be interpreted as a direct effect of imaging modality. Flap weight and the number of microvascular anastomoses performed are not systematically captured as distinct variables in this registry and could therefore not be included as covariates; perforator number, which is available, is analyzed separately as an outcome rather than as a covariate of operative time (Section 3.3). This comparison should accordingly be considered descriptive rather than a covariate-adjusted estimate of the independent effect of imaging modality on operative time.

The temporal trend observed during the observation period, with declining CTA use and increasing CCDS adoption, may reflect growing awareness of radiation exposure with CTA, typically approximately 10–15 mSv for abdominal CTA [24,34]. A randomized study by Bonde et al. reported no significant difference in operative time or complication rates between CTA- and Doppler ultrasound-guided DIEP flap surgery when adequate experience was present, supporting the feasibility of radiation-free imaging alternatives [26,29]. Concurrent secular changes in surgical experience and technique over the 2011–2018 observation period may also have contributed to the observed differences, independent of imaging modality itself.

An additional finding was the interaction between BMI and perfusion territory. While BMI had no significant effect in Holm zones 1–3, obese patients undergoing Holm zone 4 reconstructions showed substantially increased major complication rates. Although based on a relatively small subgroup, this finding suggests that obesity may become clinically relevant, particularly in extended perfusion territories. Because the present analyses were exploratory and not fully adjusted for procedural complexity, these findings should be interpreted cautiously. This subgroup finding is based on a comparatively small number of flaps including Holm zone 4, was not corrected for multiple testing, and should therefore be regarded as exploratory and hypothesis-generating rather than confirmatory.

These findings have direct clinical implications. Preoperative CTA-based mapping may facilitate selective perforator choice without compromising safety. Routine inclusion of multiple perforators does not appear to reduce major complication risk. In patients with obesity requiring extended flap designs, careful preoperative counselling and consideration of intraoperative perfusion assessment, such as indocyanine green angiography, may be warranted.

The present study has several important strengths. It represents one of the largest multicenter analyses examining the influence of preoperative perforator mapping modality in DIEP flap breast reconstruction and reflects contemporary real-world practice across a nationwide network of specialized microsurgical centers. The registry design enabled evaluation of multiple imaging modalities across a large number of flaps, including subgroup analyses according to unilateral versus bilateral reconstruction and Holm perfusion territories. Furthermore, the study links imaging strategy with operative decision-making, perforator selection, and clinical outcomes within a standardized multicenter dataset.

Several limitations should be acknowledged. As a retrospective multicenter registry analysis, differences in institutional practice patterns, case selection, and surgeon experience may have contributed to residual confounding. Imaging modality choice was not randomized and likely reflects routine clinical practice. Consequently, imaging modality may have been influenced by institutional preferences, temporal changes in practice, surgeon experience, local availability of imaging techniques, and individual patient or procedural characteristics. Although these factors may have contributed to residual confounding, the multicenter design reflects contemporary real-world practice. In addition, the registry did not capture detailed perforator characteristics or intraoperative decision-making, limiting perforator-specific analysis. The relatively small MRA subgroup and exploratory BMI–Holm interaction analyses should therefore be interpreted cautiously. Finally, multivariable adjustment for all potential confounders was not feasible within the available registry structure. Potential unmeasured confounders—including center preference, surgeon experience, case complexity, and patient characteristics—may have influenced both modality choice and outcomes. In addition, the registry period (2011–2018) predates some recent refinements in duplex and CT protocols; although the compared modalities remain in routine clinical use today, findings should be interpreted as reflecting the comparative relationship between modalities during this timeframe rather than the absolute performance of current-generation devices.

## 5. Conclusions

This large multicenter registry analysis demonstrates that preoperative mapping modality significantly influences both perforator selection and postoperative major complication rates in DIEP flap breast reconstruction. CTA-guided planning was associated with the fewest included perforators and the lowest observed major complication rate. These findings support the use of individualized anatomy-informed perforator selection strategies. The absolute number of included perforators was not predictive of major complications when stratified by perfusion territory. Obesity appears to increase complication rates in extended perfusion territories. These findings support individualized preoperative planning and suggest that high-BMI patients considered for Holm zone 4 DIEP flap reconstruction should receive detailed risk counselling and may benefit from enhanced intraoperative perfusion assessment.

## Figures and Tables

**Table 1:** Preoperative perforator mapping device, surgical duration, mean number of included perforators, and major complications in DIEP flap breast reconstruction (n = 3691 surgeries; 4283 flaps). Values are mean ± SD. Perforators: mean number of perforators included per flap; for unilateral DIEP one value is given, for bilateral DIEP values for side 1 and side 2 are shown separately. Major complications = total or partial flap loss. For perforator analysis, one implausible data entry (n = 1, perforator count = 244) was excluded. Assignment to diagnostic group by hierarchy: MRA > CTA > CCDS > HHDS > None. Total and partial flap loss are reported separately in the two rightmost columns and sum to the major complications count in every row; partial flap loss here reflects only losses meeting the major-complication threshold (a further 29 flaps registry-wide had a documented partial loss below this threshold and are not included). Percentages are calculated per flap (for bilateral subgroups, against 2 × n, since flap loss is recorded per breast even though n reflects the number of operations).

Mapping Device	Subgroup	Flaps (n)	Duration (min, Mean ± SD)	Perforators (Mean ± SD)	Major Complications n (%)	Total Flap Loss n (%)	Partial Flap Loss n (%)
None	unilateral	629	220.3 ± 79.7	2.09 ± 0.72	22 (3.5%)	18 (2.9%)	4 (0.6%)
	bilateral	107	303.3 ± 96.1	2.05 ± 0.60/2.06 ± 0.58	10 (4.7%)	7 (3.3%)	3 (1.4%)
HHDS	unilateral	691	289.4 ± 104.4	2.16 ± 1.01	26 (3.8%)	12 (1.7%)	14 (2.0%)
	bilateral	122	393.8 ± 130.6	2.11 ± 0.94/2.05 ± 0.87	12 (4.9%)	8 (3.3%)	4 (1.6%)
CCDS	unilateral	424	276.9 ± 99.3	2.62 ± 0.90	21 (5.0%)	15 (3.5%)	6 (1.4%)
	bilateral	104	408.2 ± 102.0	2.27 ± 0.70/2.20 ± 0.77	8 (3.8%)	5 (2.4%)	3 (1.4%)
CTA	unilateral	1266	317.8 ± 97.3	1.71 ± 0.85	23 (1.8%)	15 (1.2%)	8 (0.6%)
	bilateral	246	444.4 ± 142.5	1.80 ± 0.97/1.76 ± 1.00	8 (1.6%)	7 (1.4%)	1 (0.2%)
MRA	unilateral	52	330.1 ± 63.2	1.46 ± 0.92	3 (5.8%)	1 (1.9%)	2 (3.8%)
	bilateral	12	376.8 ± 114.7	1.50 ± 0.67/1.58 ± 1.00	1 (4.2%)	0 (0.0%)	1 (4.2%)
Total	all flaps	4283	304.5 ± 116.2	1.99 ± 0.93	134 (3.1%)	88 (2.1%)	46 (1.1%)
	unilateral	3099	285.9 ± 102.3	2.00 ± 0.94	95 (3.1%)	61 (2.0%)	34 (1.1%)
	bilateral	1184	400.7 ± 134.9	1.98 ± 0.88/1.94 ± 0.89	39 (3.3%)	27 (2.3%)	12 (1.0%)

**Table 2:** Number of DIEP flaps registered in each year used for analysis. Simultaneous bilateral flaps were counted double for total flap count.

Year	Total (n)	Unilateral (n)	Simultaneous Bilateral (n)
2011	204	162	21
2012	613	437	88
2013	518	396	61
2014	607	469	69
2015	688	458	115
2016	795	569	113
2017	602	416	93
2018	256	192	32
Total	4283	3099	592

**Table 3:** Patient demographics and cohort characteristics.

Variable	Value
Age (mean)	51.1 years (SD ± 9.5; range 21–87)
BMI kg/m^2^ (mean)	26.1 kg/m^2^ (SD ± 4.21; range 18.5–46.7)
Normal (18.5–24.9)	45.6%
Overweight (25.0–29.9)	36.9%
Obese (≥30.0)	17.5%
Operative time—unilateral	285.9 min (SD ± 102.1; range 95–872 min)
Operative time—bilateral	400.1 min (SD ± 134.9; range 142–1041 min)

**Table 4:** (n = 3691 surgeries) (2011–2018, DIEP flaps only). Assignment by hierarchy: MRA > CTA > CCDS > HHDS > None. Missing values excluded (n = 38), resulting in n = 3653 with valid data. The distribution was comparable in both unilateral and bilateral subgroups.

Year	None n (%)	HHDS n (%)	CCDS n (%)	CTA n (%)	MRA n (%)	n Valid
2011	2 (1.1%)	127 (72.2%)	1 (0.6%)	46 (26.1%)	0 (0.0%)	176
2012	170 (32.9%)	83 (16.1%)	48 (9.3%)	215 (41.7%)	0 (0.0%)	516
2013	141 (31.2%)	72 (15.9%)	35 (7.7%)	199 (44.0%)	5 (1.1%)	452
2014	26 (4.9%)	197 (36.8%)	75 (14.0%)	231 (43.1%)	7 (1.3%)	536
2015	65 (11.4%)	142 (24.8%)	88 (15.4%)	259 (45.3%)	18 (3.1%)	572
2016	165 (24.3%)	69 (10.2%)	101 (14.9%)	322 (47.5%)	21 (3.1%)	678
2017	164 (32.3%)	75 (14.8%)	83 (16.3%)	173 (34.1%)	13 (2.6%)	508
2018	3 (1.4%)	48 (22.3%)	97 (45.1%)	67 (31.2%)	0 (0%)	215
Total	736 (20.1%)	813 (22.3%)	528 (14.5%)	1512 (41.4%)	64 (1.8%)	3653

**Table 5:** In none of the three groups was the difference in perforator count between cases with and without major complications statistically significant. Notably, in Groups 1 and 3 (Holm 1–2 and Holm 1–4), the mean perforator count was slightly higher in the major complication group, though this trend did not reach significance. A total of 50 cases were excluded due to missing Holm zone or perforator data (n = 3049 analyzed).

Group	n	Major Complications	Mean Perforators (No Major Compl.)	Mean Perforators (Major Compl.)	U	*p*
Holm 1–2	445	13 (2.9%)	2.15 ± 1.01	2.31 ± 0.75	3.230	0.331
Holm 1–3	2354	69 (2.9%)	1.95 ± 0.88	1.90 ± 0.96	74.020	0.355
Holm 1–4	250	10 (4.0%)	2.43 ± 1.01	2.70 ± 1.49	1.292	0.663

## Data Availability

Data can be made available on demand.

## References

[1] Robert Koch-Institute (2023). Brustdrüse. Krebs in Deutschland.

[2] Gerber B., Marx M., Untch M., Faridi A. (2015). Breast Reconstruction Following Cancer Treatment. Dtsch. Arztebl. Int..

[3] Lang J.E., Summers D.E., Cui H., Carey J.N., Viscusi R.K., Hurst C.A., Waer A.L., Ley M.L.B., Sener S.F., Vijayasekaran A. (2013). Trends in post-mastectomy reconstruction: A SEER database analysis. J. Surg. Oncol..

[4] Albornoz C.R., Bach P.B., Mehrara B.J., Disa J.J., Pusic A.L., McCarthy C.M., Cordeiro P.C., Matros E. (2013). A paradigm shift in U.S. breast reconstruction: Increasing implant rates. Plast. Reconstr. Surg..

[5] Alderman A.K., Hawley S.T., Morrow M., Salem B., Hamilton A., Graff J.J., Katz S. (2011). Receipt of delayed breast reconstruction after mastectomy: Do women revisit the decision?. Ann. Surg. Oncol..

[6] Jeong W., Lee S., Kim J. (2018). Meta-analysis of flap perfusion and donor site complications for breast reconstruction using pedicled versus free TRAM and DIEP flaps. Breast.

[7] Knox A.D.C., Ho A.L., Leung L., Tashakkor A.Y., Lennox P.A., Van Laeken N., Macadam S.A. (2016). Comparison of Outcomes following Autologous Breast Reconstruction Using the DIEP and Pedicled TRAM Flaps: A 12-Year Clinical Retrospective Study and Literature Review. Plast. Reconstr. Surg..

[8] Blondeel P.N. (1999). One hundred free DIEP flap breast reconstructions: A personal experience. Br. J. Plast. Surg..

[9] Koshima I., Soeda S. (1989). Inferior epigastric artery skin flaps without rectus abdominis muscle. Br. J. Plast. Surg..

[10] Eisenhardt S.U., Momeni A., von Fritschen U., Horch R.E., Stark G.B., Bannasch H., Harder Y., Heitmann C., Kremer T., Rieger U.M. (2018). Breast reconstruction with the free TRAM or DIEP flap—What is the current standard? Consensus Statement of the German Speaking Working Group for Microsurgery of the Peripheral Nerves and Vessels. Handchir. Mikrochir. Plast. Chir..

[11] Lee B.T., Agarwal J.P., Ascherman J.A., Caterson S.A., Gray D.D., Hollenbeck S.T., Khan S.A., Loeding L.D., Mahabir R.C., Miller A.S. (2017). Evidence-Based Clinical Practice Guideline: Autologous Breast Reconstruction with DIEP or Pedicled TRAM Abdominal Flaps. Plast. Reconstr. Surg..

[12] Macadam S.A., Zhong T., Weichman K., Papsdorf M., Lennox P.A., Hazen A., Matros E., Disa J., Mehrara B., Pusic A.L. (2016). Quality of Life and Patient-Reported Outcomes in Breast Cancer Survivors: A Multicenter Comparison of Four Abdominally Based Autologous Reconstruction Methods. Plast. Reconstr. Surg..

[13] Zhong T., Novak C.B., Bagher S., Maass S.W.M.C., Zhang J., Arad U., O’nEill A.C., Metcalfe K.A., Hofer S.O.P. (2014). Using propensity score analysis to compare major complications between DIEP and free muscle-sparing TRAM flap breast reconstructions. Plast. Reconstr. Surg..

[14] Erdmann-Sager J., Wilkins E.G.M., Pusic A.L.M., Qi J.M., Hamill J.B., Kim H.M.S., Guldbrandsen G.E.B., Chun Y.S. (2018). Complications and Patient-Reported Outcomes after Abdominally Based Breast Reconstruction: Results of the Mastectomy Reconstruction Outcomes Consortium Study. Plast. Reconstr. Surg..

[15] Andrades P., Fix R.J., Danilla S., Howell R.E., Campbell W.J., De la Torre J., Vasconez L.O. (2008). Ischemic complications in pedicle, free, and muscle sparing transverse rectus abdominis myocutaneous flaps for breast reconstruction. Ann. Plast. Surg..

[16] Fritschen U.V., Grill B., Wagner J., Schuster H., Sukhova I., Giunta R.E., Heitmann C., Andree C., Horch R.E., Kneser U. (2020). Quality assurance in breast reconstruction—Establishment of a prospective national online registry for microsurgical breast reconstructions. Handchir. Mikrochir. Plast. Chir..

[17] Prantl L., Moellhoff N., Fritschen U.V., Germann G., Giunta R.E., Zeman F., Kehrer A., Lonic D., Broer P.N., Ehrl D. (2020). Impact of Smoking Status in Free Deep Inferior Epigastric Artery Perforator Flap Breast Reconstruction: A Multicenter Study. J. Reconstr. Microsurg..

[18] Prantl L., Moellhoff N., Von Fritschen U., Giunta R.E., Germann G., Kehrer A., Lonic D., Zeman F., Broer P.N. (2020). Immediate versus secondary DIEP flap breast reconstruction: A multicenter outcome study. Arch. Gynecol. Obstet..

[19] Heidekrueger P.I., Moellhoff N., Horch R.E., Lohmeyer J.A., Marx M., Heitmann C., Fansa H., Geenen M., Gabka C.J., Handstein S. (2021). Overall Complication Rates of DIEP Flap Breast Reconstructions in Germany—A Multi-Center Analysis Based on the DGPRAC Prospective National Online Registry for Microsurgical Breast Reconstructions. J. Clin. Med..

[20] Heidekrueger P.I., Fritschen U., Moellhoff N., Germann G., Giunta R., Zeman F., Prantl L. (2021). Impact of body mass index on free DIEP flap breast reconstruction: A multicenter cohort study. J. Plast. Reconstr. Aesthetic Surg..

[21] Heidekrueger P., von Fritschen U., Moellhoff N., Germann G., Giunta R., Zeman F., Prantl L. (2022). Comparison of venous couplers versus hand-sewn technique in 4577 cases of DIEP-flap breast reconstructions—A multicenter study. Microsurgery.

[22] Prantl L., Moellhoff N., von Fritschen U., Giunta R., Germann G., Kehrer A., Thiha A., Ehrl D., Zeman F., Broer P.N. (2021). Effect of Radiation Therapy on Microsurgical Deep Inferior Epigastric Perforator Flap Breast Reconstructions: A Matched Cohort Analysis of 4577 Cases. Ann. Plast. Surg..

[23] Colakoglu S., Tebockhorst S., Freedman J., Douglass S., Siddikoglu D., Chong T.W., Mathes D.W. (2022). CT angiography prior to DIEP flap breast reconstruction: A randomized controlled trial. J. Plast. Reconstr. Aesthetic Surg..

[24] Fitzgerald O’Connor E., Rozen W.M., Chowdhry M., Band B., Ramakrishnan V.V., Griffiths M. (2016). Preoperative computed tomography angiography for planning DIEP flap breast reconstruction reduces operative time and overall complications. Gland Surg..

[25] Rozen W.M., Palmer K.P., Suami H.M., Pan W.R., Ashton M.W.F., Corlett R.J.F., Taylor G.I. (2008). The DIEA branching pattern and its relationship to perforators: The importance of preoperative computed tomographic angiography for DIEA perforator flaps. Plast. Reconstr. Surg..

[26] Bajus A., Kubek T., Dražan L., Veselý J., Novák A., Berkeš A., Streit L. (2023). Surgeon-conducted color Doppler ultrasound deep inferior epigastric artery perforator mapping: A cohort study and learning curve assessment. J. Plast. Reconstr. Aesthetic Surg..

[27] Alonso-Burgos A., García-Tutor E., Bastarrika G., Cano D., Martínez-Cuesta A., Pina L. (2006). Preoperative planning of deep inferior epigastric artery perforator flap reconstruction with multislice-CT angiography: Imaging findings and initial experience. J. Plast. Reconstr. Aesthetic Surg..

[28] Bhullar H., Hunter-Smith D.J., Rozen W.M. (2020). Fat Necrosis After DIEP Flap Breast Reconstruction: A Review of Perfusion-Related Causes. Aesthetic Plast. Surg..

[29] Klasson S., Svensson H., Malm K., Wassélius J., Velander P. (2015). Preoperative CT angiography versus Doppler ultrasound mapping of abdominal perforator in DIEP breast reconstructions: A randomized prospective study. J. Plast. Reconstr. Aesthetic Surg..

[30] Malhotra A., Chhaya N., Nsiah-Sarbeng P., Mosahebi A. (2013). CT-guided deep inferior epigastric perforator (DIEP) flap localization—Better for the patient, the surgeon, and the hospital. Clin. Radiol..

[31] Mulvey C.L., Cooney C.M., Daily F.F.B., Colantuoni E., Ogbuago O.U., Cooney D.S., Rad A.N., Manahan M.A., Rosson G.D., Sacks J.M. (2013). Increased Flap Weight and Decreased Perforator Number Predict Fat Necrosis in DIEP Breast Reconstruction. Plast. Reconstr. Surg. Glob. Open.

[32] Wong C., Saint-Cyr M., Mojallal A., Schaub T., Bailey S.H., Myers S., Brown S., Rohrich R.J. (2010). Perforasomes of the DIEP flap: Vascular anatomy of the lateral versus medial row perforators and clinical implications. Plast. Reconstr. Surg..

[33] Rupra R.S., Ruccia F., Daneshi K., Aftab F., Yousif Y.F., Khan G.R., Dehnadi S., AlSaidi Y.H., Maggialetti N., Lorusso G. (2025). A systematic review and meta-analysis on computed tomography angiography mapping for deep inferior epigastric perforator flap breast reconstruction. Front Oncol..

[34] D’Angelo A., Cina A., Macrì G., Belli P., Mercogliano S., Barbieri P., Grippo C., Franceschini G., D’archi S., Mason E.J. (2021). Conventional CT versus Dedicated CT Angiography in DIEP Flap Planning: A Feasibility Study. J. Pers. Med..

